# Galaxy Dnpatterntools for Computational Analysis of Nucleosome Positioning Sequence Patterns †

**DOI:** 10.3390/ijms23094869

**Published:** 2022-04-28

**Authors:** Erinija Pranckeviciene, Sergey Hosid, Indiras Maziukas, Ilya Ioshikhes

**Affiliations:** 1 Department of Human and Medical Genetics, Biomedical Science Institute, Vilnius University, LT-08661 Vilnius, Lithuania; indiras.maziukas@stud.mf.vu.lt; 2 Department of Systems Analysis, Faculty of Informatics, Vytautas Magnus University, LT-44404 Kaunas, Lithuania; 3 All-Russia Research Institute for Agricultural Microbiology, 196608 Saint Petersburg, Russia; sergey.hosid@gmail.com; 4 Department of Biochemistry, Microbiology and Immunology, Faculty of Medicine, University of Ottawa, Ottawa, ON K1H 8M5, Canada; 5 Ottawa Institute of Systems Biology (OISB), Ottawa, ON K1H 8M5, Canada

**Keywords:** nucleosome, nucleosome positioning sequence (NPS) pattern, chromatin structure, Galaxy

## Abstract

Nucleosomes are basic units of DNA packing in eukaryotes. Their structure is well conserved from yeast to human and consists of the histone octamer core and 147 bp DNA wrapped around it. Nucleosomes are bound to a majority of the eukaryotic genomic DNA, including its regulatory regions. Hence, they also play a major role in gene regulation. For the latter, their precise positioning on DNA is essential. In the present paper, we describe Galaxy dnpatterntools—software package for nucleosome DNA sequence analysis and mapping. This software will be useful for computational biologists practitioners to conduct more profound studies of gene regulatory mechanisms.

## 1. Introduction

Nucleosomes in the genome provide measures of packaging and epigenetic layer of information to guide interactions of trans-acting proteins with the genome and regulate access to the functional elements of the genome by their positioning [1]. Information about the occurrence of a nucleosome along DNA is encoded in certain features of the sequence [2,3,4,5]. Nucleosome-favoring DNA sequences are characterized by specific 10–10.4 base pairs periodical compositions of AA/TT and CC/GG dinucleotides [6] and references therein. The sequence features [3] together with other factors such as transcription factor binding and remodeling complexes play role in nucleosome positioning in vivo [7]. Nucleosomes take part in chromatin activity and their positioning and occupancy at a global genome wide level impact genome operation. Biological consequences of nucleosome positioning and occupancy vary between cell types and conditions [8]. Core histone sequences are conserved among different species, therefore the biophysical principles of the histone assembly that determine histone preferences to certain DNA sequences should be universal across organisms [9].

### 1.1. Features of Nucleosomal DNA Sequences

It is thought that certain nucleosomes [10] in genomes are positioned by a preference of some DNA sequence patterns over the other. In vitro, certain features of the DNA sequence have much higher affinity to the histone octamer such as Widom 601 sequence [2]. Within 147 bp that are wrapped around the histone octamer a periodical recurrence of distinctive dinucleotides facilitates a sharp bending of DNA around the nucleosome [11]. It is known that specific compositions of dinucleotides make DNA more bendable [6,12]. The nucleosome linker regions show strong preference to sequences that resist DNA bending and disfavor nucleosome formation [13] and the GC rich nucleosome regions have higher nucleosome density while AT regions are more nucleosome depleted [14].

Intrinsic histone preferences of specific k-mer sequences might be species specific [15] and ensembles of nucleosomal DNA can differ between species and in turn the nucleosome use in gene regulation [10]. In higher eukaryotes genomic elements are closed by nucleosome but in unicellular organisms the genomic sites are open unless a nucleosome is repositioned there. Promoters of multicellular organisms are characterized by sequences favoring nucleosomes and in unicellular organisms by the disfavoring sequences [16]. Statistically, dinucleotide patterns in nucleosomal DNA provide information about histones sequence preferences in forming nucleosomes that are packing DNA.

### 1.2. Patterns of Dinucleotide Distributions in the Ensembles of Nucleosomal DNA Sequences

Usually, the patterns of the dinucleotide distributions in nucleosomal DNA are derived from an ensemble or in other words a “bulk” of sequences of the best phased +1 nucleosomes in the genome. The +1 nucleosome is one of primary factors determining how the rest of the nucleosomes will assemble. Sequence based mechanisms governing dynamics of nucleosome positioning and stability across variety of different conditions can be revealed through a statistical analysis of nucleosomal DNA sequences [6,17].

Recently performed comparison of patterns of dinucleotide distribution in the nucleosomal DNA in higher human and mouse organisms and in unicellular yeast organism superimposed on the superhelical locations (SHL) revealed clear regularities [17]. The SHL comprise minor and major grooves in nucleosomal DNA and were derived by Cui and Zhurkin from roll angles of crystal structures of nucleosome core particle (NCP) [12]. It was shown that the WW (weak-weak W = A or T) dinucleotide peaks in nucleosomal DNA in normal human CD4+ cells and in mouse nucleus accumbens cells (NAC) coincide and are located in the major grove SHL zones while the SS (strong-strong S = C or G) dinucleotide peaks are located in the minor grove SHL zones. The patterns of the WW and SS dinucleotide distributions in human apoptotic cells were found to be very similar to the same patterns in yeast and inverse to the patterns in human and mouse. The WW and SS maximum peaks in human and mouse correspond to the SS and WW maximum peaks of yeast and human apoptotic cells. Similarly, the RR/YY (R = A or G / Y = C or T) dinucleotide patterns in human and mouse seem to be shifted by 4–5 base pair step from the RR/YY patterns in yeast. The RR and YY in all three organisms alternate in 3 to 5 base pair steps and again, the RR peaks in human and mouse occur in major grove SHL zones, while in yeast they are in minor groove SHL zones.

**Figure 1:**
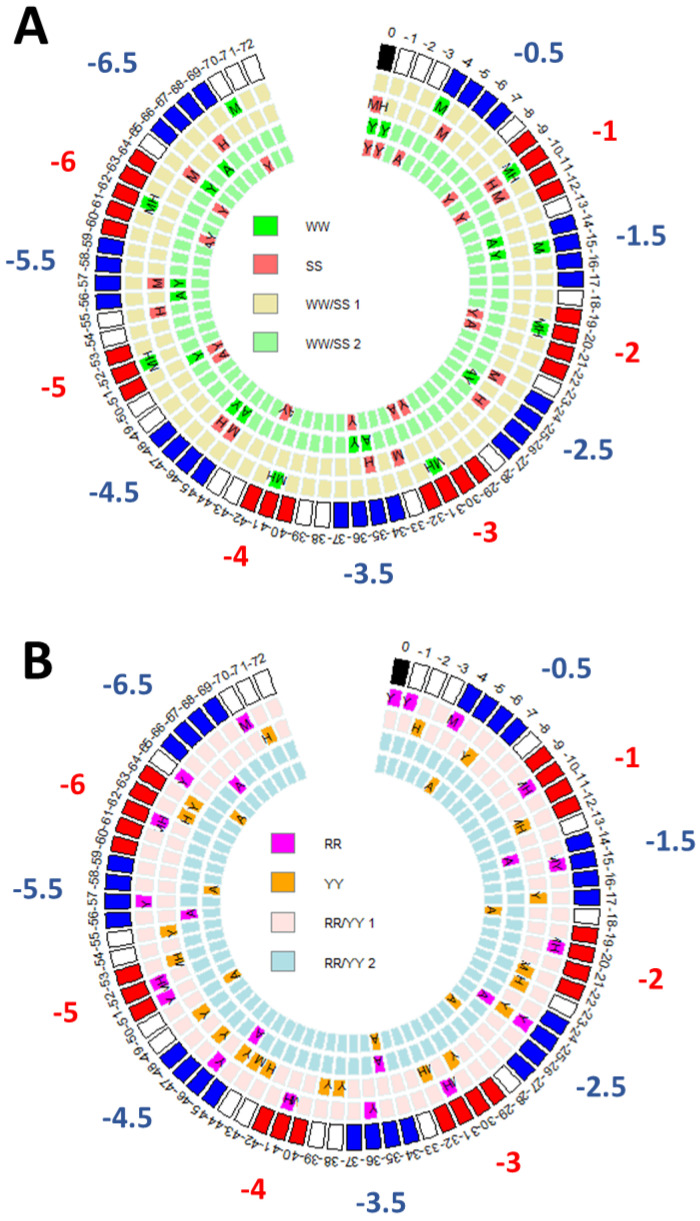
Distribution of WW/SS (Panel (**A**)) and RR/YY (Panel (**B**)) peaks in human, mouse and yeast across one half of a nucleosomal DNA represented as a circular diagram of 1 base pair steps. Peaks are indicated by letters in cells and each cell represents 1 base pair. The outer circles indicate major (red) and minor (blue) grove superhelical locations (SHL). A black cell represents a nucleosome’s dyad position. Capital letters in cells denote the organism and condition: Label A denotes human apoptotic cells, M denotes mouse nucleus accumbens cells, H denotes human CD4+ cells and Y denotes yeast. The dinucleotides—WW peaks are green, SS peaks are red, RR peaks are magenta and YY are orange. The WW/SS 1 stands for a pattern; the WW/SS 2 stands for anti-pattern and similarly for RR/YY. On the circular diagram the pattern and anti–pattern occupy two rows each representing incompatible dinucleotides.

The existence of the patterns in which WW/SS and RR/YY dinucleotides are used in opposite ways termed pattern and anti-pattern was described in [6]. Such patterns of alternating peaks of WW/SS and RR/YY dinucleotides in DNA sequences of nucleosomes were further characterized in [17] in human and mouse cells. Figure 1 shows the arrangement of WW/SS and RR/YY peaks of the said patterns in human, mouse and yeast on a circular diagram [18] using the schema of a nucleosome core particle (NCP) published by Cui and Zhurkin [12] as a guide. The WW/SS and RR/YY peaks mostly are found at specific SHL positions. The incompatible dinucleotides (the WW and SS, and similarly, the RR and YY) in different organisms and cell types occur at a very close proximity. This hints about a possible universal structure of nucleotide arrangements in nucleosome positioning sequences that is partially agnostic to a dinucleotide identity [19].

### 1.3. Computational Tools

To analyse genome-wide positioning and occupancy of nucleosomes and to derive patterns of dinucleotide distributions in NPS from a bulk of the nucleosomal DNA sequences the specialized computational tools must be available.

Usually, a nucleosome occupancy and positioning information is obtained from a coverage profile computed from an alignment of the micrococcal nuclease sequencing (MNase-Seq) reads to a reference genome of the investigated organism. The profile peak positions usually are determined using a traditional Gaussian [20] or improved wavelet smoothing [21]. If MNase-Seq data of cases and controls is available, then nucleosome positioning, shift and occupancy change events can be computed by the DANPOS software [22]. Algorithms and guidelines to determine nucleosome positioning and occupancy from MNase-Seq data are well established [23,24,25,26,27]. Newly emerging nucleosome mapping algorithms use machine learning [28]. A review of the available tools and approaches can be found in [29].

On the contrary, a software to compute patterns of dinucleotide distributions from a bulk/ensemble of nucleosomal DNA sequences and to map nucleosomes in sequences using the computed patterns is not sufficiently addressed in the scientific literature [29,30,31]. We attempt to fill this gap by contributing dnpatterntools software utilities. By contributing we mean packaging and making our developed software available for the wider community via the Galaxy framework [32] of the reproducible bioinformatics research. Our software serves as a computational platform to compute new and to reproduce previously reported patterns of dinucleotide distributions in a given bulk/ensemble of the nucleosomal DNA sequences [6,17,33]. The dnpatterntools suite is available as a package of the standalone routines and as Galaxy tools. The Galaxy tools are also available through a dockerized Galaxy from the docker hub.

## 2. Results

The dnpatterntools provide utilities to compute and analyze patterns of dinucleotide frequency distributions given a bulk/ensemble of nucleosomal DNA FASTA sequences and to map nucleosomes in the FASTA sequences given the patterns.

### 2.1. Implementation

The dnpatterntools consist of core programs and tool utilities. The core programs are written in C/C++. Some of them use a SeqAn library [34]. The SeqAn library is a collection of C++ header definitions of functions specifically written to work with genomic data (FASTA, BAM and VCF). A C++ program that uses SeqAn library depends only on the SeqAn function headers. Other utilities in dnpatterntools are shell scripts wrapped into fully functional Galaxy [32] tool and submitted to a test Galaxy Tool Shed. The core utilities are available through bioconda channel [35]. Thus enabling their integration into the Galaxy environment. The dnpatterntools include randomized shuffling of k-letters in the sequences [36] to be used in Galaxy wrapper to test null hypothesis. A Fourier program to compute periodograms of dinucleotide frequency of occurrence in NPS patterns [33] and a Mapping_CC program to map a nucleosome in a sequence by a pattern [6] that were developed as standalone programs to support publications are now integrated into the Galaxy framework. Table 1 summarizes core programs and tool utilities. See Data availability section for more details.

### 2.2. Workflow

**Figure 2:**
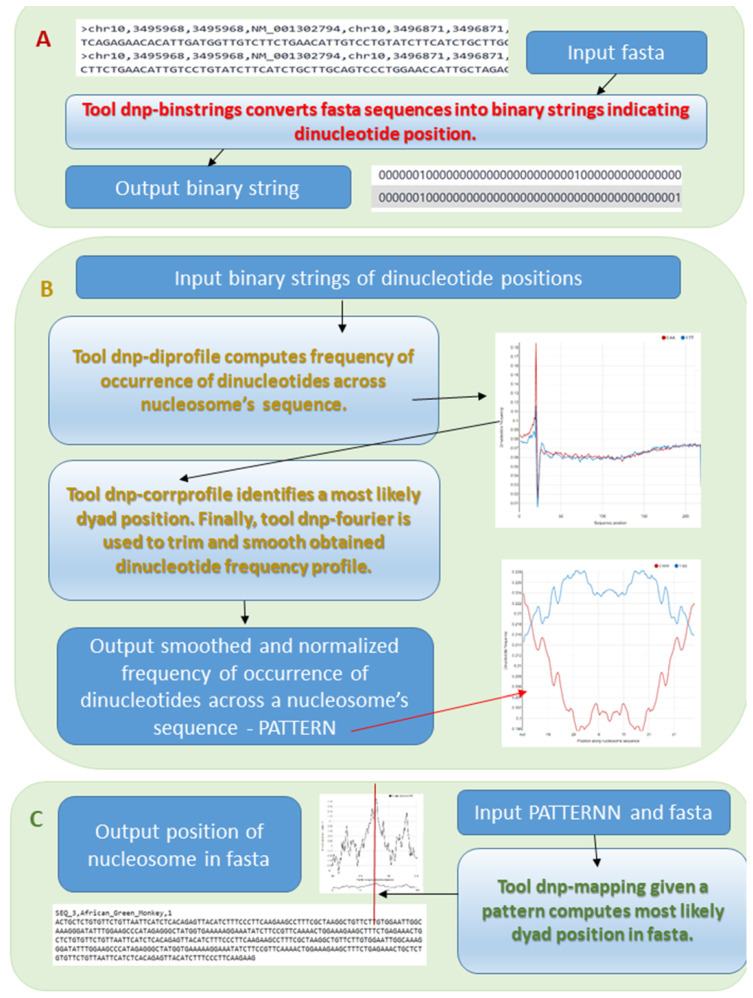
Main components of the galaxy dnpatterntools package. (Panel (**A**)) represents a conversion of nucleosome fasta sequences aligned by the experimental end into the binary representations in which 1 indicates presence of the dinucleotide for which a pattern is derived. (Panel (**B**)) represents an abstraction of a workflow to compute nucleosome positioning sequence patterns. From binary representation of the fasta sequences the frequency of the dinucleotide occurrences at each sequence position is computed. A graph on the right shows frequencies for AA and TT dinucleotides of mouse sequences from GSE54263. Big peak marks cleavage site. This profile serves as input to the tool that identifies a most likely dyad position of a nucleosome from the forward and reverse dinucleotide frequency profile. See explanations in the main text. Then the identified dinucleotide frequency profile is trimmed and smoothed resulting in a PATTERN that can be used to map ncleosome position in other fasta sequences. The right graph below shows patterns of composite dinucleotides WW and SS obtained after determining a dyad position, symmetrization and smoothing. (Panel (**C**)) shows mapping of a nucleosome position using a derived pattern in some DNA sequence.

We presented the dnpatterntools software suite in the Bioinformatics Community Conference 2020 (BCC2020) [37]. In the following we briefly review all tools and a newly added mapping tool in more detail. The main components of the galaxy dnpatterntools package are shown in Figure 2.

A basic dnpatterntools workflow to compute patterns of dinucleotide frequency distributions from nucleosome sequences consists of several steps:

1. computation of distribution of frequency of dinucleotide occurrences in a batch of aligned sequences;
2. determination of nucleosome position in the sequences;
3. selection and symmetrization of dinucleotide frequency profiles from the determined interval;
4. computation of frequency profiles of composite dinucleotides WW/SS (W = A or T and S = C or G) and RR/YY (R = A or G and Y = C or T);
5. normalization and smoothing of the frequency profiles to remove noise
6. computation of the periodograms.

This workflow was used to obtain patterns analyzed in [17] from the three datasets of nucleosome sequences to compute dinucleotide patterns: human CD4+ cells [38], apoptotic lymphocyte cells [39] and nucleus accumbens cells of mouse brain retrieved from GSE54263 [40].

We will briefly review each step of NPS patterns computation in turn.

#### 2.2.1. Computation of Distributions of Dinucleotide Frequencies along Nucleosomal DNA from a Batch of Sequences

Nucleosomal DNAs are generally obtained from the purified chromatin stabilized with formaldehyde and digested with MNnase which cleaves sequence specific linker sites [21]. However, sequence fragments resulting from MNase digestion have substantial variability from 10 to 20 bp in the precise fragment ends [5]. In dinucleotide frequency profiles computed from nucleosome sequences obtained by MNase-Seq and aligned by experimental end of a cleavage site manifests as a narrow large peak because of the sequence specificity. In profiles of dinucleotide frequencies a region of a cleavage site can be identified by a large peak at the beginning of the computed frequency profiles.

Although MNase-Seq is a most common way to obtain nucleosome’s DNA [21], other approaches exist [41]. The MNase-Seq is also affected by a transient unwrapping of nucleosomal DNA and for this reason other chemical mapping methods measuring nucleosome locations directly were developed [42,43]. Another popular in vivo nucleosome mapping method is DNase-seq that was used to produce nucleosome maps in yeast and human [44]. Lastly, a cost efficient method of nucleosome mapping albeit of less resolution is FAIRE (Formaldehyde Assisted Isolation of Regulatory Elements) [45] that is most useful in establishing chromatin profiles of diverse cell types and to probe the effects of small molecules on chromatin organization.

The patterns of dinucleotide frequencies are computed from a batch of aligned sequences of nucleosomes DNA. At each position of the nucleosome sequence a frequency of occurrence is computed for each dinucleotide. Given a binary matrix of dinucleotde occurrences in sequences coded as 1 and else as 0, a frequency profile is simply a sum of occurrences of the selected dinucleotide at every position along the sequence normalized by the number of sequences. See Step 1 in Figure 2. Patterns of dinucleotide frequency distributions represent statistical sequence-specific features of nucleosomal DNA. Patterns originating from different organisms, conditions or experimental manipulations may have signatures characteristic only to that particular condition [6].

#### 2.2.2. Determination of Nucleosome Position Using Dyad-Symmetry of Dinucleotide Frequency Profiles

Dyad-symmetry is a hallmark of the nucleosome DNA sequence [46]. The peak arrangements in patterns of dinucleotide (most often AA, TT, AT, CC, GC or GG) frequency distribution along the nucleosomal sequence have a recognizable dyad-symmetry. These dinucleotides statistically are preferred and are periodically distributed along nucleosome DNA sequence [46]. These dinucleotide preferences were investigated and reported by studies in vitro [2,47], statistically [5,48], from analysis of nucleosome stability [49] and computationally [6,50,51]. Dyad-symmetry feature helps to determine a position of a nucleosome in a batch of sequences aligned by experimental end—because at the nucleosome position centered on the dyad the forward and complementary profiles of dinucleotides will have a maximum positive correlation. See panel B in Figure 2. It shows Pearson correlation coefficient at each position along the sequence computed between forward (fw) and reversed complement (rc) of frequency profiles for selected dinucleotides within the window corresponding to the nucleosome size of 146 bp. In such obtained matrix of Pearson correlation coefficients a maximum positive correlation between of fw and rc frequency profiles of either AA, TT, TA, CC, GG and GC dinucleotides or combination will indicate a nucleosome position-same for all sequences in a batch.

#### 2.2.3. Correlations between Forward and Reverse Patterns

Identification of nucleosome position via correlation cannot be fully automated, because correlations vary in both: along the sequence and in different conditions. The position in which fw and rc frequency profiles of one or several dinucleotides attain maximum positive correlation ought to be at a close proximity to the cleavage site, a positive indicator of a nucleosome start. In mouse strongest correlations were found between fw and rc frequency profiles for AA/TT dinucleotides. However, in human cells the strongest correlations were found for AT and GC dinucleotides. Panel B of Step 2 in Figure 2 shows correlation profiles obtained for all three cases: nucleosomes in mice brain, human CD4+ cells and apoptotic lymphocyte cells. Solid dark red line represents inferred a most likely start position of the nucleosome from the dinucleotide frequency profiles.

#### 2.2.4. Patterns of Dinucleotide Frequency Distributions and Their Periodograms

Nucleosome sequences in yeast are characterized by a very clear pattern of AA/TT frequency distribution with peaks occurring each 10 base pairs [6,11]. In other organisms other dinucleotides may have stronger patterns. For example, in human, mouse and fly the GC/CG/CC/GG dinucleotide periodicity correlates better with nucleosome positioning [38,52,53]. It was also shown that patterns of composite RR/YY (purine-purine/pyrimidine-pyrimidine) dinucleotides can be associated with nucleosome stability [6]. In dnpatterntools in addition to frequency distribution of all 16 dinucleotides we also compute frequencies of composite dinucleotides strong-strong/weak-weak SS/WW (S = C or G, W = A or T) and purine-purine/pyrimidine-pyrimidine RR/YY (R = A or G, Y = C or T).

#### 2.2.5. Symmetrization

The frequency distributions on original forward sequences and their complement should be equally represented in the pattern. Therefore, for each dinucleotide its fw and rc patterns are averaged at each position. This step is called symmetrization. Finally, to improve a representation of the patterns they are smoothed by applying a moving average filter and a several positions of a pattern are trimmed from both ends to avoid a boundary effect. In practice a size of a moving average smoothing window is 3 positions and each end is trimmed by 4 positions as shown in the schematics in Figure 2.

#### 2.2.6. Periodicity of Dinucleotide Steps

A spectral decomposition of a pattern reveals the strongest periodical components of the pattern. The dinucleotide frequency distributions in nucleosome sequences are expected to have peaks at 10 bp and the periods multiple of 10. The peaks significantly expressed around 10 bp period vary across conditions and dinucleotides. Spectral decomposition may serve as means to identify a leading dinucleotide pattern in each condition. Step 3 right panel in Figure 2 shows periodograms of the patterns on the left.

#### 2.2.7. Dinucleotide Shuffling

In order to show biological relevance of the biological sequence analysis they are compared with the results that would be expected by chance. Random shuffling of nucleotides in sequences is a technique that destroys a periodical structure of dinucleotide occurrences in nucleosome sequences, but preserves sequence composition. We include a Galaxy wrapper to the uShuffle program in the dnpatterntools [36]. The uShuffle program has options to specify the lenght of k-mers (mono-, di-, tri-nucleotides) frequency of which should be preserved. In our experiments we used k = 2 (dinucleotides).

## 3. Mapping of Nucleosome Positions in Sequence by Pattern

Nucleosome positioning sequence patterns derived from a batch of nucleosomal DNA sequences aligned by the experimental end can be used to map most likely position of a nucleosome in a sequence [6]. For the first time we integrated a standalone Mapping_CC program [29,54] into the package of dnpatterntools so that it can be easily used in Galaxy to map nucleosomes in the sequences of interest.

### 3.1. Mapping Algorithm

**Figure 3:**
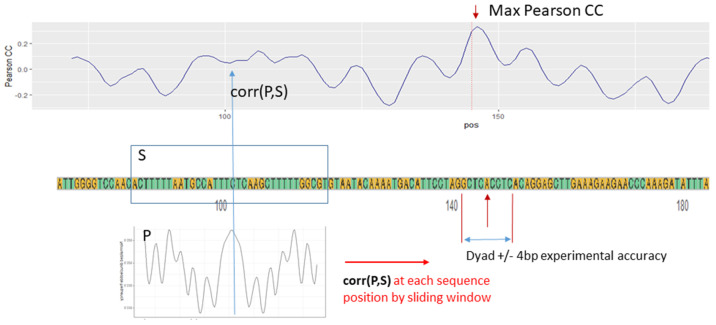
Illustration of a nucleosome mapping in a given sequence by pattern. A pattern P represents a dinucleotide frequency along a nucleosomal DNA. The dinucleotode can be either one of 16 dinucleotodes—AA, CC, GG, TT, ⋯—or one of the composite dinucleotides WW, SS, RR or YY. The pattern P of the length of the nucleosome (147 base pair positions, or slightly shortened by a trimming from both ends) and a segment of a sequence S of the same length as P are used to compute Pearson correlation coefficient. The computed Pearson correlation coefficient (CC) is reported at a sequence position corresponding to a dyad position of the pattern P and it shows how likely it is for that sequence position to be a nucleosome dyad. The Pearson CC between the pattern P and the sequence S is computed within a sliding window along the full DNA sequence as shown in a top panel. The maximum positive Pearson CC indicates a most likely nucleosome’s dyad position. In this example this position is marked by 100. For a single unique most likely position of a dyad to be identified, a whole DNA sequence of interest has to be constrained to two length of a nucleosome (less than 294 bp).

Computation of Pearson correlation between a sequence and a pattern is at a core of the Mapping_CC program. The process of computation is illustrated and described in Figure 3.

Figure 3 represents a real nucleosomal sequence from a database compiled in [55] in which a known dyad position is at a sequence position 145. For each sequence in the database the dyad position is known within the limits of experimental accuracy. In this example the accuracy is ±4 base pairs. In the example in Figure 3 the WW pattern was derived from human apoptotic cells [33]. The nucleosome dyad position identified by the algorithm in Mapping_CC is not precise, but deviates by one base pair. This example illustrates computation using only one sequence and only one pattern. In Galaxy framework the mapping procedure can be applied on multiple sequences and using multiple patterns. If using multiple patterns, then the maximum Pearson CC can be computed by averaging values of Pearson CC for each position along a sequence. However, other inference methods can be applied.

### 3.2. Mapping Application in Nucleosome DNA Sequences of 17 Organisms

We tested the Mapping_CC algorithm on sequences of nucleosomal DNA for which a position of nucleosome’s dyad is known within the limits of experimental accuracy [55]. These sequences were used in one of the earliest works showing an existence of AA/TT periodical patterns in nucleosome positioning sequence. Out of 204 sequences we selected 173 that did’t have Ns masking the nucleotides. These sequences comprise 17 organisms and are available from a supplementary data.

Previously we derived patterns in NPS [6,17,33] using sequences of nucleosomes in human CD4+ [38] and apoptotic cells [39] and MNase-Seq data of mouse brain nucleus accumbens cells [40]. These patterns are referred to by cd4, apo and mcon in this study and are available as a supplementary material of [17].

**Figure 4:**
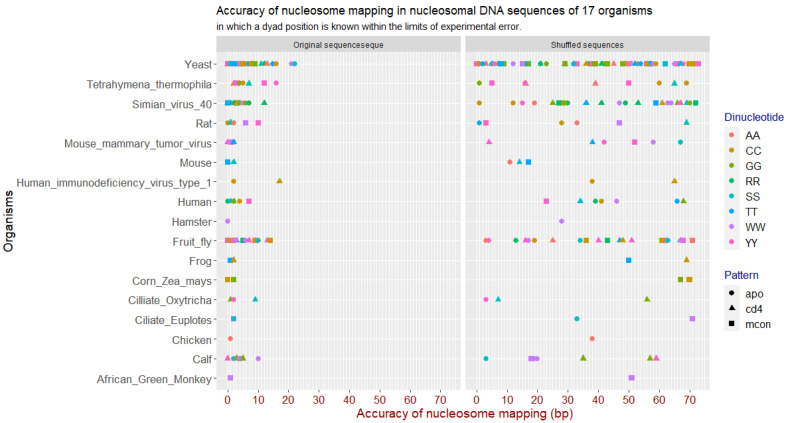
Nucleosome mapping accuracy in nucleosomal DNA sequences of 17 organisms. The x axis represents the distance in base pairs from the true dyad position and the best mapping position. The y axis shows each organism. The points on the grid represent the accuracy for each sequence. For some organisms such as yeast and fruit fly there were more sequences than for the others. The left panel shows best mapping result for the original sequence. The right panel shows mapping result using the same best mapping pattern on the shuffled sequence.

For each sequence we performed mapping with Mapping_CC using previously derived NPS patterns from mouse and human organisms using AA, CC, GG, TT, WW, SS, RR and YY dinucleotides. Since the dyad position is known in each sequence (400 bp long) to be around the position 200 within the given limits of the experimental accuracy, we were able to measure a mapping accuracy for each sequence and each pattern. The best mapping pattern for each sequence was identified by its mapping accuracy - how close it mapped the dyad with respect to the true dyad. Figure 4 shows accuracy of the best mapping patterns for each original sequence and its shuffled counterpart [36]. A frequency of dinucleotide occurrence is preserved in the shuffled sequences.

Each sequence has a best mapping pattern, that maps a nucleosome with the highest accuracy in the given sequence and using Mapping_CC we are able to determine the best mapping pattern and its accuracy for the individual sequences.

In shuffled sequences the best mapping pattern did not locate nucleosomes where they previously were, because in the shuffled sequences any position—or better to say position inside and outside the error limits—is equally likely.

Therefore we expected that in shuffled sequences the best pattern will map nucleosome at a random position, because any position is equally likely. The Figure 4 actually illustrates that. Predicted positions on the native nucleosomal sequences have trend to be close to its experimental position (Figure 4, left panel) when predicted position on the shuffled sequences have random distribution (Figure 4, right panel). Especially well it is seen for organisms, for which there are more sequences—yeast, fruit fly, simian virus 40.

## 4. Discussion

There is ample experimental evidence for the role of specific nucleosome positioning in gene regulation. General mechanism of influence of the nucleosome positioning on gene regulation is related to the chromatin compaction by phased nucleosomes, resulting in a lesser DNA availability for transcriptional machinery. The nucleosome positioning is determined by sequence and non-sequence factors, such as ATP dependent remodeling factors and transcription factors. In this paper we are focused on the software development and description for the sequence-dependent nucleosome mapping. Our methodology was proven successful in number of publications [6,17,33]. Knowing precise nucleosome location is critical for understanding how cis-regulatory elements control genetic information. Our software package will be a valuable tool for researchers studying gene regulation.

We created Galaxy wrappers for all standalone dnpatterntools software modules and integrated them into a Galaxy instance. We made the Galaxy dnpatterntools instance available via the docker hub and demonstrated its use. Interested users can make their own Galaxy instances with dnpatterntools and add other tools as all instructions are available through the GitHub. By making our tools available through Galaxy framework we offer interested users very high flexibility to use our tools in various bioinformatics projects.

A variety of sequencing protocols and technologies exist that can probe organization of a chromatin and nucleosome DNA occupancy such as Hi-C sequencing [56], ATAC-seq [57] and a single-cell MNase-seq [58]. Our tools can work with nucleosomal DNA sequences obtained by any technology as long as they are formatted as fasta sequences.

## 5. Materials and Methods

This article presents software. Therefore, the methods are considered results.

## Figures and Tables

**Table 1:** List of utilities in dnpatterntools.

Core Utilities
C/C++ binary tools (bioconda package)
Compute binary strings from sequences (dnp-binstrings).
Compute dinucleotide frequencies in sequences (dnp-diprofile).
Compute correlation between forward and reverse complement profiles (dnp-corrprofile).
Compute periodogram, normalization and smoothing (dnp-fourier).
Mapping_CC, map nucleosome by pattern in a given sequence (dnp-mapping).
**Helper Utilities**
Shell scripts
Binary strings for multiple dinucleotides.
Frequency profiles of all dinucleotides.
Correlations for all dinucleotides.
Select profiles within interval.
Composite WW/SS and RR/YY dinucleotide profiles.
Symmetrization of frequency profiles.
Smoothing by moving average.
Periodogram for all dinucleotides.
Gnuplot of selected columns.
Mapping nucleosomes in multiple FASTA sequences by multiple patterns.

## Data Availability

The mouse data presented in this study are openly available in Zenodo https://doi.org/10.5281/zenodo.3813510 (accessed on 30 March 2022). The Galaxy dnpatterntools and instructions to build the dockerized dnpatterntools Galaxy instance as well as the tutorials are available from https://github.com/erinijapranckeviciene/galaxy-dnpatterntools (accessed on 30 March 2022). The original previously published dnpatterntools software is openly available in GitHub https://github.com/erinijapranckeviciene/dnpatterntools (accessed on 30 March 2022).

## Supplementary Materials

The following supporting information can be downloaded at: https://www.mdpi.com/article/10.3390/ijms23094869/s1.

## Abbreviations

The following abbreviations are used in this manuscript:

NPS	Nucleosome Positioning Sequence
SHL	Super Helical Locations
NCP	Nucleosome Core Particle
GEO	Gene Expression Omnibus
NCBI	National Center of Biotechnology Information
NAC	Nucleus Accumbens Cells
fw	forward
rc	reverse complement
DANPOS	Dynamic analysis of nucleosome position and occupancy by sequencing
Hi-C	high-throughput chromosome conformation capture
ATAC-seq	Assay for Transposase-Accessible Chromatin with high-throughput sequencing
MNase-seq	micrococcal nuclease digestion with deep sequencing
